# Aberrant Functional Network Connectivity as a Biomarker of Generalized Anxiety Disorder

**DOI:** 10.3389/fnhum.2017.00626

**Published:** 2017-12-19

**Authors:** Jianping Qiao, Anning Li, Chongfeng Cao, Zhishun Wang, Jiande Sun, Guangrun Xu

**Affiliations:** ^1^School of Physics and Electronics, Shandong Normal University, Jinan, China; ^2^Shandong Province Key Laboratory of Medical Physics and Image Processing Technology, Shandong Normal University, Jinan, China; ^3^Institute of Data Science and Technology, Shandong Normal University, Jinan, China; ^4^Department of Radiology, Qilu Hospital of Shandong University, Jinan, China; ^5^Department of Emergency, Jinan Central Hospital Affiliated to Shandong University, Jinan, China; ^6^Department of Psychiatry, Columbia University, New York, NY, United States; ^7^School of Information Science and Engineering, Shandong Normal University, Jinan, China; ^8^Department of Neurology, Qilu Hospital of Shandong University, Jinan, China

**Keywords:** generalized anxiety disorder, brain connectivity, independent component analysis, Granger causality, classification

## Abstract

Neural disruptions during emotion regulation are common of generalized anxiety disorder (GAD). Identifying distinct functional and effective connectivity patterns in GAD may provide biomarkers for their diagnoses. This study aims to investigate the differences of features of brain network connectivity between GAD patients and healthy controls (HC), and to assess whether those differences can serve as biomarkers to distinguish GAD from controls. Independent component analysis (ICA) with hierarchical partner matching (HPM-ICA) was conducted on resting-state functional magnetic resonance imaging data collected from 20 GAD patients with medicine-free and 20 matched HC, identifying nine highly reproducible and significantly different functional brain connectivity patterns across diagnostic groups. We then utilized Granger causality (GC) to study the effective connectivity between the regions that identified by HPM-ICA. The linear discriminant analysis was finally used to distinguish GAD from controls with these measures of neural connectivity. The GAD patients showed stronger functional connectivity in amygdala, insula, putamen, thalamus, and posterior cingulate cortex, but weaker in frontal and temporal cortex compared with controls. Besides, the effective connectivity in GAD was decreased from the cortex to amygdala and basal ganglia. Applying the ICA and GC features to the classifier led to a classification accuracy of 87.5%, with a sensitivity of 90.0% and a specificity of 85.0%. These findings suggest that the presence of emotion dysregulation circuits may contribute to the pathophysiology of GAD, and these aberrant brain features may serve as robust brain biomarkers for GAD.

## Introduction

Generalized anxiety disorder (GAD) is one of the most common mental disorders characterized by excessive anxiety and worry that is not focused on a specific situation or object. The GAD is highly prevalent in the general population. Patients with GAD often suffer from a variety of anxiety-related physical symptoms like difficulty concentrating, irritability, muscle tension, and disturbed sleep which impair their quality of life and social functioning (Paulus and Stein, 2010; Liu et al., 2015b; Li Y. et al., 2016; Buff et al., 2017; Moon and Jeong, 2017). To improve the level of both basic GAD research and its clinical diagnosis, it is essential to understand the pathophysiological mechanisms of GAD and explore a valid and objective biomarker to distinguish patients with GAD from HC.

Previous brain imaging studies have revealed neural differences in GAD compared with HC in brain areas associated with the emotion dysregulation, cognitive deficits, and/or reward processing, which may be important in the pathophysiology of GAD. Individuals with GAD exhibited over-engagement of amygdala and inferior frontal gyrus during the viewing of negative images on an emotion regulation task, compared to HC (Fitzgerald et al., 2017). A reduced correlation between prediction error and activity within the ventromedial prefrontal cortex and striatum of GAD was shown on a decision making task, lead to their decision-making deficits, and excessive worry about everyday problems (White et al., 2017). Another task-based functional magnetic resonance imaging (fMRI) study has reported significantly higher activity in the hippocampus during the delayed-response working memory (WM) task for GAD patients, suggesting that neural activation patterns in patients with GAD are changed by the effect of neutral and anxiety-inducing distractors during a delayed response WM task (Moon and Jeong, 2015). In addition, larger volumes in amygdala (De Bellis et al., 2000; Schienle et al., 2011), putamen (Liao et al., 2013, 2014a), and altered gray matter volumes in the thalamus, hippocampus, insula, posterior cingulate, and superior temporal gyrus (STG) in GAD patients were reported in the structure magnetic resonance imaging (sMRI) studies (De Bellis et al., 2002; Strawn et al., 2013; Moon and Jeong, 2017). Diffusion tensor imaging (DTI) has shown that the GAD patients have increased fractional anisotropy (FA) in the amygdala (Zhang et al., 2013) and postcentral gyrus (Zhang et al., 2011), while reduced FA in bilateral uncinate fasciculus, body of corpus callosum, inferior fronto-occipital fasciculus, inferior longitudinal fasciculus, and corona radiate (Liao et al., 2014b; Wang et al., 2016).

Resting-state fMRI has been widely used to study the mechanism of brain function, especially in the clinical study, due to simple operation, no task stimulation, as well as the stable and reliable results. Resting-state fMRI studies have reported decreased functional connectivity (FC) between amygdala and prefrontal cortex in adults (Etkin et al., 2009; Hilbert et al., 2014) and the dysconnectivity has been considered as a longitudinal biomarker of symptom changes in GAD (Makovac et al., 2016). Adolescents with GAD exhibited disruptions in amygdala-based intrinsic FC networks that included regions in medial prefrontal cortex, insula, and cerebellum (Roy et al., 2013). The increased FC between the amygdala and the temporal pole was also revealed in GAD compared to HC in both eyes-open and eyes-closed resting-state fMRI (Li W. et al., 2016). The set of studies supports the brain structure abnormality and the functional deficit in GAD patients, suggesting that the neural system-related emotion regulation is dysfunctional. However, whether and how the brain neural alterations differ in individuals with GAD remains unclear.

Machine learning techniques have played an important role in exploring the brain differences between patients with anxiety disorder and HC. Recently, one study has employed resting-state functional MRI data to investigate multivariate classification of social anxiety disorder by using whole-brain FC (Liu et al., 2015a). Neural face processing in the fear network and gray matter volume alterations over the whole brain were also used to separate anxiety disorder from controls (Frick et al., 2014). These studies demonstrated that FC and structural alterations had good diagnostic potential for anxiety disorder which may be used as a complementary tool in clinical diagnosis.

Independent component analysis (ICA) is a multivariate method for blind source separation which has been widely applied for analyzing neuroimaging data (Calhoun et al., 2009). As a data-driven approach, ICA can be used to identify brain networks in resting-state fMRI without using a priori information of the source signals. Each independent component (IC) provides a grouping of brain regions that share the same pattern.

At present, there are many methods to measure the effective connectivity such as structural equation model (SEM), dynamic causal model, and Granger causality (GC). The SEM and dynamic causal model does not contain time information, which also need to select interaction region in advance. A mistake on the model assumption will lead to a wrong result. GC overcomes these limitations effectively. GC analysis is very consistent with the actual situation because it does not require any prior knowledge and considers the effect of the time on the results (Marinazzo et al., 2011; Cohen Kadosh et al., 2016).

The aim of the current study was to examine whether brain functional and effective connectivity networks differ in GAD. We applied the ICA with hierarchical partner matching (HPM-ICA) to assess the brain FC networks. The causal influence between the IC was estimated by utilizing GC method. The machine learning was finally used to distinguish the GAD from HC by combining the features of functional and effective connectivity brain networks. We hypothesized that we would detect brain connectivity differences in GAD within the cortical–subcortical neural systems that support emotion dysregulation and these feature patterns could be served as biomarkers for GAD.

## Materials and Methods

### Participants

Twenty participants with GAD who met the criteria for DSM-IV (13 females, 7 males, mean age 41.5 ± 10.7 years, range 30–50 years) were recruited from the psychological outpatient clinic at the Qilu Hospital of Shandong University. Twenty group matched by age and sex HC (13 females, 7 males, mean age 40.1 ± 9.8 years, range 30–49 years) were recruited by public advertisement to take part in the study. All GAD participants had been diagnosed by a licensed psychiatrist before enrolment. All participants were right-handed, native Chinese speakers. Exclusion criteria were: age younger than 18 years, lifetime history of increased intracranial pressure, seizure disorder, stroke, brain tumor, multiple sclerosis or brain surgery, cognitive impairment, history of substance-use disorder, an active autoimmune, endocrine, vascular disorder affecting the brain, any unstable cardiac disease, hypertension, and severe renal, liver insufficiency. All patients and controls were medication free for at least 1 month before enrolment. The safety screening form and informed consent form were approved by the Institutional Review Board of Qilu Hospital of Shandong University. We obtained written informed consent from all participants.

Psychiatric diagnoses were assessed using the Structured Clinical Interview for DSM-IV (SCID-I). We evaluated anxiety severity on the day of MRI scan using the assessment of the Hamilton Rating Scale for Anxiety (Hamilton, 1959; Deshpande et al., 2010). Anxiety severity ranged from mild to severe, with an average severity of 19.10 ± 5.68 for GAD. The average HAMA score of HC is 0.65 ± 0.77. The Hamilton Rating Scale for Depression (HAMD) (Hamilton, 1967) was also administered. The mean HAMD score of GAD is 7.86 ± 3.22 and the mean HAMD score of HC is 0.71 ± 0.90. All participants were informed of the safety and eligibility criteria for fMRI scanning: no neurological conditions and no implanted ferrous metal.

### Image Acquisition

Images were acquired on a Siemens Verio 3.0 Tesla MRI scanner (Siemens, Erlangen, Germany) using a 32-channel head coil at the Qilu Hospital of Shandong University. We used earplugs to reduce scanner noise and restraining foam pads to reduce head motion. Participants were instructed to rest with their eyes closed but not to fall asleep during scanning. When the scanning process was completed, we asked all the participants whether they were asleep during scanning. In addition, the participants had involuntary movement of body if they were asleep, leading to the motion artifact of fMRI data. The two rules controlled the participants who fall asleep during scanning were excluded in this study. Resting-state fMRI data were acquired using a single-shot gradient-echo planar imaging (EPI) sequence. Thirty-six contiguous axial slices were acquired along the AC–PC plane, with a 64 × 64 matrix and 20 cm field of view (voxel size = 3.4375 × 3.4375 × 3.0 mm^3^, repetition time = 2000 ms, echo time = 30 ms, flip angle = 90 degrees, slice thickness = 3 mm). The sequence took 8 min, resulting in a total of 240 volumes.

### Image Analysis

#### HPM-ICA-Based Functional Connectivity

Firstly, we preprocessed the resting-state fMRI images by using SPM12 (Welcome Department of Imaging Neuroscience, London, United Kingdom) that was run under MATLAB. The functional scans were slice timing-corrected, spatially realigned to the first scan to correct for head movements, normalized to the Montreal Neurological Institute (MNI) coordinate system and spatially smoothed using an isotropic 8 mm full-width at half-maximum (FWHM) Gaussian kernel.

Secondly, we applied the ICA with HPM-ICA, which we proposed and published previously (Wang et al., 2011; Qiao et al., 2015, 2017), to the preprocessed fMRI data to estimate the brain FC networks. In this method, we used ICA to generate *N* ICs for each participant and then used partner matching to match these components across the participants, yielding clusters of components that matched across participants. For each of these clusters, we ran one-sample *t*-tests to generate cluster maps. The main limitation of simple partner matching is that it assumes a fixed number *N* of IC, but that number is not known *a priori*, and it may even vary across participants. We therefore used a hierarchical approach that includes a second level of partner matching. In this second level, we used various numbers of components (from 20 to 120 with increments of 10). For each of these sets of component numbers, we performed the first-level partner matching to generate the cluster maps. We then applied partner matching again across these cluster maps (the second level), to determine which clusters mapped across sets containing differing numbers of components. This hierarchical procedure yielded clusters of clusters. For each cluster of clusters, we then selected the cluster that had the highest Cronbach’s alpha for further analyses. In this way, we identified eight reproducible clusters across all the participants.

Finally, group-level statistical analysis was applied to detect random effects of group difference in FC between GAD and HC. We entered the *z*-score maps of the eight clusters of ICs into a second-level factorial analysis, covarying for age and sex. All the statistical contrasts were corrected for multiple comparisons to *p* < 0.01 by enforcing a minimum cluster extent of 30 voxels. The main idea of the cluster extent threshold method is to model the entire imaging volume, assume an individual voxel type I error, smooth the volume with a Gaussian kernel, and then count the number of voxel clusters of each size. After a number of iterations, a probability associated with each cluster extent is calculated, and the cluster extent threshold yielding the desired correction for multiple comparisons can be enforced (Slotnick et al., 2003; Slotnick, 2017). The cluster size was obtained by using custom software written in MATLAB (Slotnick et al., 2003). We ran 10,000 Monte Carlo simulations based on individual voxel. In a single simulation, we smoothed the activation map by using a three-dimensional 8-mm FWHM Gaussian kernel. The Pearson’s correlation analysis was also performed in GAD patients to investigate the correlation between the anxiety severity (HAMA score) and brain connectivity.

#### Granger Causality-Based Effective Connectivity

Granger causality was applied to estimate the effective connectivity or causal influences between the IC that were identified using the HPM-ICA method. The time courses of these components were used to compute GC indices (GCIs) between them. We then used two-sample *t*-tests to detect group difference in GCIs between GAD and HC.

#### Feature Selection and Classification

A feature selection technique is often performed before classification to avoid the curse of dimensionality and enhance generalization of the classifier. The central premise when using feature selection is that the data contain some features that are either redundant or irrelevant, and can thus be removed without incurring much loss of information. In this study the functional and effective connectivity were considered together to serve as features and two-sample *t*-tests were performed to determine the features that showed differences between the GAD and HC. Features with significant differences (*p* < 0.05, uncorrected) between the two groups of training set were selected. We used two classifiers to distinguish GAD from HC which were the maximum uncertainty linear discriminant analysis (MLDA) classifier (Dai et al., 2012) and a kernel support vector machine (SVM) in which a Gaussian kernel with width of 0.5 was used. A leave-one-out cross-validation (LOOCV) strategy was used to evaluate the performance of a classifier. The feature selection was carried out only on the training set of each LOOCV fold which avoided the introduction of bias.

We also assessed the classification contribution of each feature in the MLDA classifier by using the coefficients of the discrimination hyperplane which measured the weights of the features. The selected features were consistent in each iteration of LOOCV, but feature weights were based on a slightly different subset of the data. Therefore, the final feature weights were the average across all folds of LOOCV.

## Results

### Reproducible Independent Components

**FIGURE 1:**
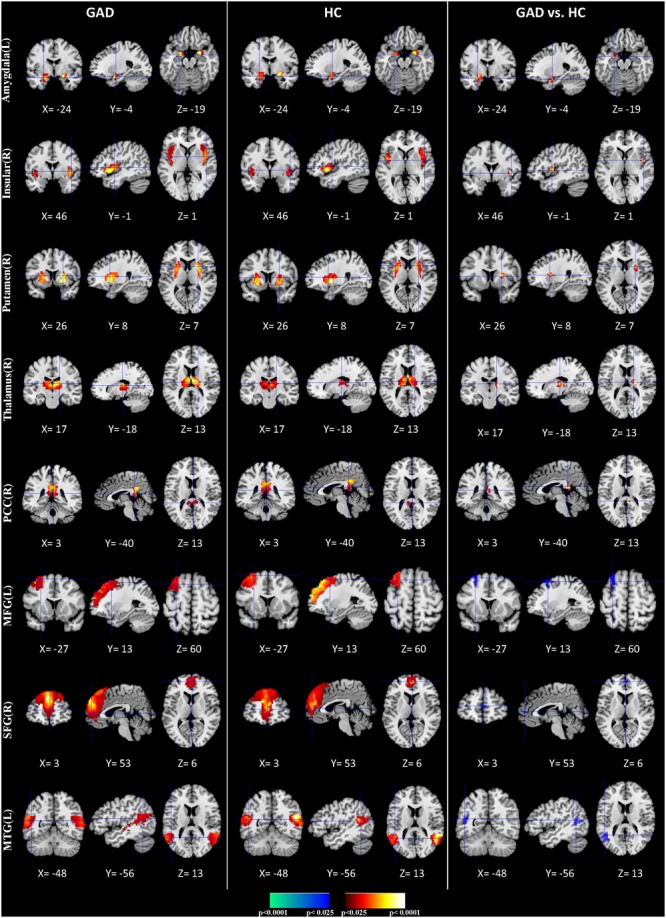
Comparisons of FC between GAD and HC. The first three columns display the random-effect group activity maps detected from the GAD. The first column is a coronal view, the second is a sagittal view, and the third is an axial view. The second three columns display the group activity maps detected from the HC. Each row displays one group activity map generated by applying a one-sample *t*-test to one of the eight clusters of IC. Any two group activity maps within the same row across the first three and second three columns are significantly similar to one another in their spatial configurations. The last three columns display *t*-contrast maps comparing the group activity maps from the GAD and control participants. The images show that FC was greater in GAD compared with HC participants in the amygdala, insular, putamen, thalamus, and posterior cingulate cortex (PCC) (shown in red), but their FC was weaker in the middle frontal gyrus (MFG), Superior frontal gyrus (SFG), and MTG (shown in blue).

**FIGURE 2:**
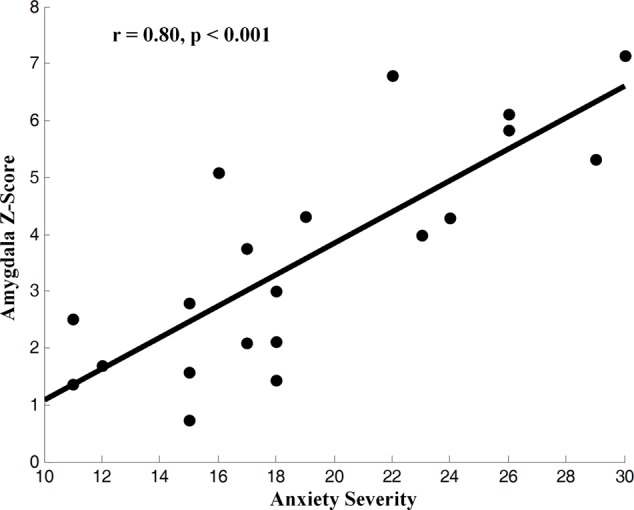
Correlation of *z*-score for blood-oxygen-level-dependent (BOLD) activity, an fMRI index of neural activity, in the IC from the left amygdala with the severity of anxiety in GAD (*z*-transformed total score in the measures). BOLD correlated with symptom severity, indicating that greater neural activity accompanied greater severe symptoms across GAD participants.

We applied HPM-ICA to identify eight clusters of IC that were significantly reproducible in their spatial patterns across GAD and control participants. The general linear model in SPM was used to perform a one-sample *t*-test on each of the eight clusters to generate eight random-effect IC maps that represented statistically significant FC (**Figure 1**). Then we compared the eight ICs of GAD and HC in a second-level, random-effects analysis. We generated eight masks from all GAD and HC corresponding to each IC or resting-state network (RSN) from one-sample *t*-tests results. The group comparisons were restricted or masked to the voxels within corresponding resting-state networks masks (Sorg et al., 2007; Mohammadi et al., 2009; Liao et al., 2010). Compared with controls, GAD showed significantly stronger connectivity in the left amygdala, right insula, right putamen, right posterior cingulate cortex (PCC), right thalamus, while weaker FC in the left middle frontal gyrus (MFG), right superior frontal gyrus (SFG), and left middle temporal gyrus (MTG) (**Table 1** and **Figure 1**). The anxiety severity (HAMA score) had a significant positive correlation with the FC in the left amygdala in GAD participants (*r* = 0.80, *p* < 0.001) (**Figure 2**).

**Table 1:** Regional locations and significant comparisons of the independent component maps between GAD and healthy controls.

Brain areas	Location		Peak location			*T-*statistic
	Side	BA	*x*	*y*	*z*	
**GAD vs. HC (positive)**						
Amygdala	L	NA	–24	–4	–19	+2.94
Insula	R	16	46	–1	1	+2.72
Putamen	R	NA	26	8	7	+2.65
Thalamus	R	NA	17	–18	13	+2.34
Posterior cingulate cortex (PCC)	R	23	3	–40	13	+3.19
**GAD vs. HC (negative)**						
Middle frontal gyrus (MFG)	L	9	–27	13	60	–3.23
Superior frontal gyrus (SFG)	R	8	3	53	6	–2.97
Middle temporal gyrus (MTG)	L	21	–48	–56	13	–3.06

*All coordinates are in the MNI ICBM152 space. NA, not applicable.*

### Granger Causality Interactions

**FIGURE 3:**
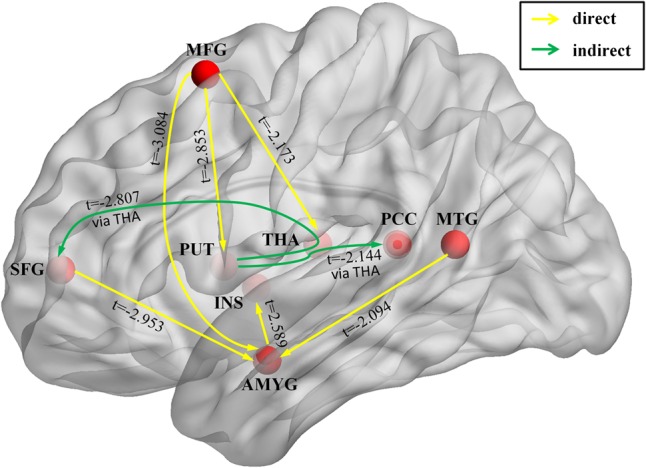
Brain circuit that involved in emotion dysregulation in GAD. The image shows the significant interregional causal connections as estimated by the Granger causality index (GCI) and the comparison of GCIs between the GAD and HC. Yellow lines represent causal influences from region *X* to the region *Y*. Green lines represent causal influence from region *X* to region *Y* via the thalamus. The arrowhead shows the direction of each causal influence. The *t*-score means the group difference in GCIs between GAD and HC. Only significant connections are shown. Abbreviations: AMYG, amygdala; INS, insula; PUT, putamen; THA, thalamus; PCC, posterior cingulate cortex; MFG, middle frontal gyrus; SFG, superior frontal gyrus; MTG, middle temporal gyrus.

We used GCIs to assess the effective connectivity between the IC involved in emotion dysregulation brain networks, including amygdala, insula, putamen, thalamus, PCC, MFG, SFG, and MTG. Basal ganglia are involved in many neuronal pathways including emotional, motivational, associative, and cognitive functions. The striatum (caudate nucleus, putamen, and nucleus accumbens) receive inputs from all cortical areas (top-down) and, throughout the thalamus, project principally to frontal lobe areas (bottom-up) (Herrero et al., 2002). Therefore, two types of causality indices were produced: the connectivity from region A to B (top-down), the connectivity from region A to B through region C, where C indicated activity in the thalamus when assessing causal influences from the basal ganglia to the cortices (bottom-up) (Wang et al., 2011). The effective connectivity was weaker in GAD than in HC in connections from the MFG to amygdala, from MTG to amygdala, from the MFG to putamen, and from the putamen to SFG via the thalamus (**Table 2** and **Figure 3**).

**Table 2:** Comparisons of statistically significant GCIs of the interregional connections of the reproducible IC.

	GAD	HC	GAD vs. HC
MFG → Amygdala	0.125 ± 0.054, *p* = 4.95*E*-08	0.225 ± 0.108, *p* = 1.50*E*-03	*t* = -3.084, *p* = 5.40*E*-03
SFG → Amygdala	0.120 ± 0.063, *p* = 7.48*E*-07	0.224 ± 0.108, *p* = 1.60*E*-03	*t* = -2.953, *p* = 7.40*E*-03
MTG → Amygdala	0.061 ± 0.046, *p* = 5.14*E*-05	0.141 ± 0.147, *p* = 4.36*E*-02	*t* = -2.094, *p* = 4.80*E*-02
Amygdala → Insula	0.071 ± 0.049, *p* = 2.50*E*-05	0.021 ± 0.013, *p* = 6.60*E*-03	*t* = 2.589, *p* = 1.68*E*-02
MFG → Putamen	0.039 ± 0.023, *p* = 3.19*E*-06	0.083 ± 0.055, *p* = 7.20*E*-03	*t* = -2.853, *p* = 9.30*E*-03
SFG → Putamen	0.031 ± 0.034, *p* = 1.80*E*-03	0.067 ± 0.032, *p* = 1.50*E*-03	*t* = -2.320, *p* = 3.00*E*-02
MFG → Thalamus	0.187 ± 0.060, *p* = 7.51*E*-10	0.276 ± 0.144, *p* = 2.30*E*-03	*t* = -2.173, *p* = 4.08*E*-02
Putamen → PCC via Thalamus	0.017 ± 0.028, *p* = 2.45*E*-02	0.066 ± 0.086, *p* = 9.01*E*-02	*t* = -2.144, *p* = 4.33*E*-02
Putamen → SFG via Thalamus	0.027 ± 0.027, *p* = 8.97*E*-04	0.084 ± 0.073, *p* = 2.35*E*-02	*t* = -2.807, *p* = 1.03*E*-02

*The data in each cell in the second and third columns represent the mean ± std of the GCIs. We used two-sample *t*-test to compare the GCIs of GAD and HC, yielding the significance test statistics in the fourth column (*p* < 0.05, uncorrected). Thalamus *X* →*Y* represents the connectivity between *X* and *Y* via the thalamus.*

### Machine Learning

**FIGURE 4:**
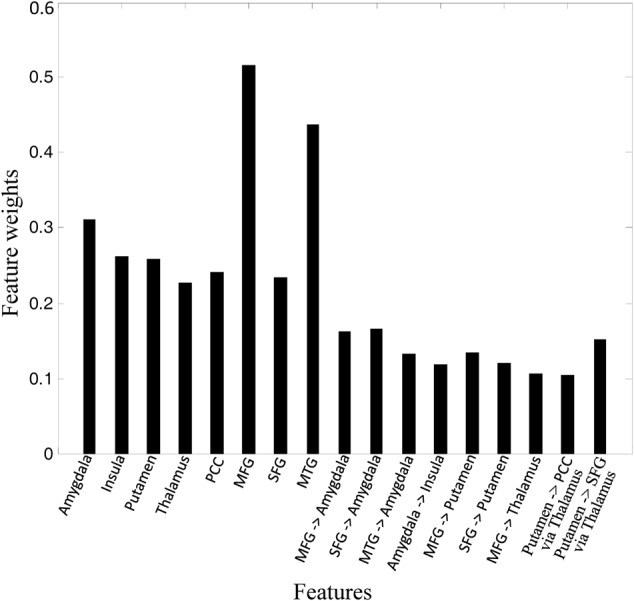
Feature weights in the classification.

We performed MLDA classifier with ICs and GCIs features, achieving a classification accuracy of 87.5%, with a sensitivity of 90.0% and a specificity of 85.0%. The weights of the features were shown in **Figure 4**. It can be seen that the connectivity in MFG, MTG, and amygdala made the largest classification contributions. We also performed kernel SVM to classify the GAD and HC, yielding an average accuracy of 92.5% with the LOOCV method. As the linear discriminant analysis does not work well when features are not linearly separable, the kernel SVM performs better than MLDA classifier by projecting features into a higher dimensional space where mapping features exhibit linear patterns.

## Discussion

In the present study, we applied HPM-ICA and GC methods on resting-state fMRI data to investigate the intrinsic differences in functional and effective connectivity between GAD and HC. Machine learning was then used to assess if these brain features can serve as biomarkers for GAD. We found that GAD subjects showed stronger FC in the amygdala, insula, putamen, PCC, and thalamus, while weaker FC in the prefrontal cortex and MTG. GC influences were generally weaker in GAD than in controls in connections from the prefrontal cortex to amygdala and subcortical regions. The results indicated that abnormal functional and effective connectivity in emotion-related brain networks was related to the generic risk of GAD, which makes it a potential endophenotype for GAD.

The amygdala plays a primary role in the processing of emotional reactions in humans and other animals (Davis and Whalen, 2001; Tye et al., 2011; Qin et al., 2014; Adhikari et al., 2015). The amygdala is the core of the coordination of autonomic and fear response (Shin and Liberzon, 2010). When the threat from outside is perceived, the amygdala is activated and projects the information into the cortex or subcortical nuclei to control arousal and autonomic responses. The amygdala may integrate all the information and then respond to the threat to activate fear circuit, leading to faster breathing, heart rate, and high blood pressure (Linares et al., 2012). Therefore, the amygdala is essential for the pathophysiology of anxiety disorders. The hyperactivation of the amygdala is reported in most anxiety disorders (Etkin and Wager, 2007; Linares et al., 2012; Li Y. et al., 2016). Consistent with previous studies, we identified that GAD patients exhibit increased FC in amygdala compared with HC, indicating that the sensitivity of the amygdala may be involved in the development of anxiety disorders.

The insula is marked as an important component of salience network (Menon and Uddin, 2010; Paulus and Stein, 2010; Cauda et al., 2011; Linares et al., 2012; Nieuwenhuys, 2012; Pannekoek et al., 2013; Peterson et al., 2014). The salience network is responsible for monitoring the individual internal and external conflicts and errors, and transmitting them to the frontoparietal control network to solve the conflict or reduce errors. Our results showed increased FC in insula, as well as increased effective connectivity from amygdala to insula, in the GAD group compared with the HC group, consistent with previous resting state (Roy et al., 2013) and task-related studies (Mcclure et al., 2007). The increased brain connectivity of insula in GAD may lead to lower thresholds for individual adaptation to the environment. That is, it is easier for GAD than HC to regard the neutral or non-conflict stimulus as threatening stimulus, resulting in the abnormal activation of anxiety circuit.

The GAD patients exhibited decreased connectivity between the prefrontal cortex and amygdala, which replicates previous neuroimaging findings (Monk et al., 2008; Etkin et al., 2009; Roy et al., 2013; Hilbert et al., 2014; Makovac et al., 2016). The prefrontal cortex is responsible for the cognitive control. Reduced connectivity of the prefrontal cortex in GAD patients has been associated with the top-down emotional dysregulation and attention deficit, which may be closely related to anxiety symptoms and anxiety characteristics. The decreased modulation function of the prefrontal cortex to amygdala may result in the abnormal continuous activation of the fear loop. Therefore, the cognitive impairment in GAD patients may lead to the clinical symptoms such as persistent anxiety and worry.

We also found decreased connectivity between the MTG and the amygdala in GAD patients compared with HC. Evidence has shown that the medial temporal lobe and its interactions with the amygdala are critically implicated in enhancing memory for intrinsically threatening stimuli (Labar and Cabeza, 2006). Moreover, emotionally arousing stimuli exert their beneficial effect on episodic memory by enhancing activity in both the amygdala and the medial temporal lobe memory system in the human brain (Dolcos et al., 2004). In addition, changes in heart rate variability over time were reported as positively correlated with the FC between amygdala and MTG in GAD (Makovac et al., 2016). Taken together, impairments in connectivity in the MTG-associated memory retrieval and attention brain network may be related to the difficult extinction of fear conditioning, leading to the continuous worry, anxiety, and apprehension for GAD patients.

We found robust and reproducible increased connectivity in thalamus in patients with GAD, which is the central core in cortical–subcortical neurocircuitry. The thalamus is an important relay station for sensory information transmission and has widely spread functional connections with cortical and subcortical areas in the brain. Previous neuroimaging studies have suggested that the thalamus plays an important role not only in the filtering of sensory information, but also involved in the process of senior cognition and emotion regulation (Herrero et al., 2002; Boatman and Kim, 2006; Haber and Calzavara, 2009). Previous findings indicate elevated activity in the thalamus in GAD patients relative to HC during directed threat imagery (Buff et al., 2017). Furthermore, increased connectivity between amygdala and thalamus was predicted by the amplified physiological responses to the induction (Makovac et al., 2016). Thus, abnormal connectivity of the thalamus may cause sensory information filtering disorder and excessive alertness in GAD patients.

The putamen showed the expected pattern of increased FC in GAD patients compared with HC. Previous studies have observed increased amygdala FC with the putamen (Liu et al., 2015c) and significantly larger gray matter volume of the putamen in GAD patients compared to HC (Liao et al., 2013). Adolescents with GAD showed putamen hyperactivation in response to valence decisions (Guyer et al., 2012). The main function of the putamen is to regulate movements and influence various types of learning. Alterations in the putamen might be associated with somatic anxiety symptoms of GAD.

Additionally, we found increased connectivity in PCC which is the central core of the default mode network (DMN). The DMN is most commonly shown to be active during passive rest and mind-wandering (Buckner et al., 2008). A previous fMRI study identified the increased FC within DMN associated with both anxiety and depression and the connectivity was positively correlated with anxiety and depression scores (Coutinho et al., 2016). Several studies have shown that intrinsic activity within PCC is altered and associated with worry in anxiety disorders (Paulesu et al., 2010; Sylvester et al., 2012; Makovac et al., 2016). Increased connectivity in PCC within the DMN in GAD patients indicated the enhancement of the processing and collection of the surrounding information which may be responsible for the tension and worry of GAD even in the resting state.

Lastly, high classification accuracy with features of altered connectivity in amygdala, insula, prefrontal cortex, temporal cortex, thalamus, and putamen could across verify that pathological change of these regions may be the neural substrates underlying the occurrence of GAD. To our knowledge, this is the first study to show that GAD can be accurately classified from HC based on functional resting-state biomarkers. In addition, the future work will focus on how to improve the classification performance by larger sample size and incorporating information from different imaging modalities.

The current study has several limitations. First, the *t*-test-based feature selection method was used in this study which does not take into account interactions between multiple features and spatial patterns. However, applications of *t*-tests in feature selection are computationally fast and easy to implement and scale well to high dimensional data. Therefore, they are able to select a small subset of relevant features from the original high-dimensional feature set. Second, we used data-driven-based analysis in this study which may lead to an incomplete biomarker set of GAD. Further study is needed to combine the ROI-based analysis and multivariate feature selection methods to find more complete and robust biomarkers of GAD.

## Ethics Statement

This study was carried out in accordance with the recommendations of DSM-IV, the Institutional Review Board of Qilu Hospital of Shandong University with written informed consent from all subjects. All subjects gave written informed consent in accordance with the Declaration of Helsinki. The protocol was approved by the Institutional Review Board of Qilu Hospital of Shandong University.

## Author Contributions

Conceived and designed the experiments: JQ, AL, and GX. Performed the experiments: JQ, AL, and GX. Analyzed the data: JQ, ZW, and JS. Contributed reagents/materials/analysis tools: JQ and ZW. Wrote the paper: JQ, ZW, CC, and GX.

## Conflict of Interest Statement

The authors declare that the research was conducted in the absence of any commercial or financial relationships that could be construed as a potential conflict of interest.

## References

[B1] Adhikari A. Lerner T. N. Finkelstein J. Pak S. Jennings J. H. Davidson T. J. (2015). Basomedial amygdala mediates top-down control of anxiety and fear. *Nature* 527 179–185. 10.1038/nature15698 26536109 PMC4780260

[B2] Boatman J. A. Kim J. J. (2006). A thalamo-cortico-amygdala pathway mediates auditory fear conditioning in the intact brain. *Eur. J. Neurosci.* 24 894–900. 10.1111/j.1460-9568.2006.04965.x 16930417

[B3] Buckner R. L. Andrews-Hanna J. R. Schacter D. L. (2008). The brain’s default network: anatomy, function, and relevance to disease. *Ann. N. Y. Acad. Sci.* 1124 1–38. 10.1196/annals.1440.011 18400922

[B4] Buff C. Schmidt C. Brinkmann L. Gathmann B. Tupak S. Straube T. (2017). Directed threat imagery in generalized anxiety disorder. *Psychol. Med.* 10.1017/S0033291717001957 [Epub ahead of print]. 28735579

[B5] Calhoun V. D. Liu J. Adali T. (2009). A review of group ICA for fMRI data and ICA for joint inference of imaging, genetic, and ERP data. *Neuroimage* 45 S163–S172. 10.1016/j.neuroimage.2008.10.057 19059344 PMC2651152

[B6] Cauda F. D’agata F. Sacco K. Duca S. Geminiani G. Vercelli A. (2011). Functional connectivity of the insula in the resting brain. *Neuroimage* 55 8–23. 10.1016/j.neuroimage.2010.11.049 21111053

[B7] Cohen Kadosh K. Luo Q. De Burca C. Sokunbi M. O. Feng J. Linden D. E. J. (2016). Using real-time fMRI to influence effective connectivity in the developing emotion regulation network. *Neuroimage* 125 616–626. 10.1016/j.neuroimage.2015.09.070 26475487 PMC4692450

[B8] Coutinho J. F. Fernandesl S. V. Soares J. M. Maia L. Goncalves O. F. Sampaio A. (2016). Default mode network dissociation in depressive and anxiety states. *Brain Imaging Behav.* 10 147–157. 10.1007/s11682-015-9375-7 25804311

[B9] Dai Z. Yan C. Wang Z. Wang J. Xia M. Li K. (2012). Discriminative analysis of early Alzheimer’s disease using multi-modal imaging and multi-level characterization with multi-classifier (M3). *Neuroimage* 59 2187–2195. 10.1016/j.neuroimage.2011.10.003 22008370

[B10] Davis M. Whalen P. J. (2001). The amygdala: vigilance and emotion. *Mol. Psychiatry* 6 13–34. 10.1038/sj.mp.4000812 11244481

[B11] De Bellis M. D. Casey B. J. Dahl R. E. Birmaher B. Williamson D. E. Thomas K. M. (2000). A pilot study of amygdala volumes in pediatric generalized anxiety disorder. *Biol. Psychiatry* 48 51–57. 10.1016/S0006-3223(00)00835-0 10913507

[B12] De Bellis M. D. Keshavan M. S. Shifflett H. Iyengar S. Dahl R. E. Axelson D. A. (2002). Superior temporal gyrus volumes in pediatric generalized anxiety disorder. *Biol. Psychiatry* 51 553–562. 10.1016/S0006-3223(01)01375-0 11950457

[B13] Deshpande G. Li Z. Santhanam P. Coles C. D. Lynch M. E. Hamann S. (2010). Recursive cluster elimination based support vector machine for disease state prediction using resting state functional and effective brain connectivity. *PLOS ONE* 5:e14277. 10.1371/journal.pone.0014277 21151556 PMC3000328

[B14] Dolcos F. Labar K. S. Cabeza R. (2004). Interaction between the amygdala and the medial temporal lobe memory system predicts better memory for emotional events. *Neuron* 42 855–863. 10.1016/S0896-6273(04)00289-2 15182723

[B15] Etkin A. Prater K. E. Schatzberg A. F. Menon V. Greicius M. D. (2009). Disrupted amygdalar subregion functional connectivity and evidence of a compensatory network in generalized anxiety disorder. *Arch. Gen. Psychiatry* 66 1361–1372. 10.1001/archgenpsychiatry.2009.104 19996041 PMC12553334

[B16] Etkin A. Wager T. D. (2007). Functional neuroimaging of anxiety: a meta-analysis of emotional processing in PTSD, social anxiety disorder, and specific phobia. *Am. J. Psychiatry* 164 1476–1488. 10.1176/appi.ajp.2007.07030504 17898336 PMC3318959

[B17] Fitzgerald J. M. Phan K. L. Kennedy A. E. Shankman S. A. Langenecker S. A. Klumpp H. (2017). Prefrontal and amygdala engagement during emotional reactivity and regulation in generalized anxiety disorder. *J. Affect. Disord.* 218 398–406. 10.1016/j.jad.2017.05.013 28501740 PMC6608590

[B18] Frick A. Gingnell M. Marquand A. F. Howner K. Fischer H. Kristiansson M. (2014). Classifying social anxiety disorder using multivoxel pattern analyses of brain function and structure. *Behav. Brain Res.* 259 330–335. 10.1016/j.bbr.2013.11.003 24239689 PMC3888925

[B19] Guyer A. E. Choate V. R. Detloff A. Benson B. Nelson E. E. Perez-Edgar K. (2012). Striatal functional alteration during incentive anticipation in pediatric anxiety disorders. *Am. J. Psychiatry* 169 205–212. 10.1176/appi.ajp.2011.11010006 22423352 PMC3307369

[B20] Haber S. N. Calzavara R. (2009). The cortico-basal ganglia integrative network: the role of the thalamus. *Brain Res. Bull.* 78 69–74. 10.1016/j.brainresbull.2008.09.013 18950692 PMC4459637

[B21] Hamilton M. (1959). The assessment of anxiety states by rating. *Br. J. Med. Psychol.* 32 50–55. 10.1111/j.2044-8341.1959.tb00467.x 13638508

[B22] Hamilton M. (1967). Development of a rating scale for primary depressive illness. *Br. J. Soc. Clin. Psychol.* 6 278–296. 10.1111/j.2044-8260.1967.tb00530.x 6080235

[B23] Herrero M. T. Barcia C. Navarro J. M. (2002). Functional anatomy of thalamus and basal ganglia. *Childs Nerv. Syst.* 18 386–404. 10.1007/s00381-002-0604-1 12192499

[B24] Hilbert K. Lueken U. Beesdo-Baum K. (2014). Neural structures, functioning and connectivity in Generalized Anxiety Disorder and interaction with neuroendocrine systems: a systematic review. *J. Affect. Disord.* 158 114–126. 10.1016/j.jad.2014.01.022 24655775

[B25] Labar K. S. Cabeza R. (2006). Cognitive neuroscience of emotional memory. *Nat. Rev. Neurosci.* 7 54–64. 10.1038/nrn1825 16371950

[B26] Li W. Cui H. Zhu Z. Kong L. Guo Q. Zhu Y. (2016). Aberrant functional connectivity between the amygdala and the temporal pole in drug-free generalized anxiety disorder. *Front. Hum. Neurosci.* 10:549. 10.3389/fnhum.2016.00549 27867352 PMC5095112

[B27] Li Y. Meng Y. Yuan M. Zhang Y. Ren Z. Zhu H. (2016). Therapy for adult social anxiety disorder: a meta-analysis of functional neuroimaging studies. *J. Clin. Psychiatry* 77 e1429–e1438. 10.4088/JCP.15r10226 27680692

[B28] Liao M. Yang F. Zhang Y. He Z. Song M. Jiang T. (2013). Childhood maltreatment is associated with larger left thalamic gray matter volume in adolescents with generalized anxiety disorder. *PLOS ONE* 8:e71898. 10.1371/journal.pone.0071898 23951265 PMC3741188

[B29] Liao M. Yang F. Zhang Y. He Z. Su L. Li L. (2014a). Lack of gender effects on gray matter volumes in adolescent generalized anxiety disorder. *J. Affect. Disord.* 155 278–282. 10.1016/j.jad.2013.10.049 24262640

[B30] Liao M. Yang F. Zhang Y. He Z. Su L. Li L. (2014b). White matter abnormalities in adolescents with generalized anxiety disorder: a diffusion tensor imaging study. *BMC Psychiatry* 14:41. 10.1186/1471-244X-14-41 24528558 PMC3937009

[B31] Liao W. Chen H. Feng Y. Mantini D. Gentili C. Pan Z. (2010). Selective aberrant functional connectivity of resting state networks in social anxiety disorder. *Neuroimage* 52 1549–1558. 10.1016/j.neuroimage.2010.05.010 20470894

[B32] Linares I. M. Trzesniak C. Chagas M. H. Hallak J. E. Nardi A. E. Crippa J. A. (2012). Neuroimaging in specific phobia disorder: a systematic review of the literature. *Rev. Bras. Psiquiatr.* 34 101–111. 10.1016/S1516-4446(12)70017-X 22392396

[B33] Liu F. Guo W. Fouche J. P. Wang Y. Wang W. Ding J. (2015a). Multivariate classification of social anxiety disorder using whole brain functional connectivity. *Brain Struct. Funct.* 220 101–115. 10.1007/s00429-013-0641-4 24072164

[B34] Liu F. Zhu C. Wang Y. Guo W. Li M. Wang W. (2015b). Disrupted cortical hubs in functional brain networks in social anxiety disorder. *Clin. Neurophysiol.* 126 1711–1716. 10.1016/j.clinph.2014.11.014 25534495

[B35] Liu W. J. Yin D. Z. Cheng W. H. Fan M. X. You M. N. Men W. W. (2015c). Abnormal functional connectivity of the amygdala-based network in resting-state FMRI in adolescents with generalized anxiety disorder. *Med. Sci. Monit.* 21 459–467. 10.12659/MSM.893373 25673008 PMC4335563

[B36] Makovac E. Watson D. R. Meeten F. Garfinkel S. N. Cercignani M. Critchley H. D. (2016). Amygdala functional connectivity as a longitudinal biomarker of symptom changes in generalized anxiety. *Soc. Cogn. Affect. Neurosci.* 11 1719–1728. 10.1093/scan/nsw091 27369066 PMC5091683

[B37] Marinazzo D. Liao W. Chen H. Stramaglia S. (2011). Nonlinear connectivity by Granger causality. *Neuroimage* 58 330–338. 10.1016/j.neuroimage.2010.01.099 20132895

[B38] Mcclure E. B. Monk C. S. Nelson E. E. Parrish J. M. Adler A. Blair R. J. (2007). Abnormal attention modulation of fear circuit function in pediatric generalized anxiety disorder. *Arch. Gen. Psychiatry* 64 97–106. 10.1001/archpsyc.64.1.97 17199059

[B39] Menon V. Uddin L. Q. (2010). Saliency, switching, attention and control: a network model of insula function. *Brain Struct. Funct.* 214 655–667. 10.1007/s00429-010-0262-0 20512370 PMC2899886

[B40] Mohammadi B. Kollewe K. Samii A. Krampfl K. Dengler R. Munte T. F. (2009). Changes of resting state brain networks in amyotrophic lateral sclerosis. *Exp. Neurol.* 217 147–153. 10.1016/j.expneurol.2009.01.025 19416664

[B41] Monk C. S. Telzer E. H. Mogg K. Bradley B. P. Mai X. Louro H. M. (2008). Amygdala and ventrolateral prefrontal cortex activation to masked angry faces in children and adolescents with generalized anxiety disorder. *Arch. Gen. Psychiatry* 65 568–576. 10.1001/archpsyc.65.5.568 18458208 PMC2443697

[B42] Moon C. M. Jeong G. W. (2015). Functional neuroanatomy on the working memory under emotional distraction in patients with generalized anxiety disorder. *Psychiatry Clin. Neurosci.* 69 609–619. 10.1111/pcn.12295 25781332

[B43] Moon C. M. Jeong G. W. (2017). Abnormalities in gray and white matter volumes associated with explicit memory dysfunction in patients with generalized anxiety disorder. *Acta Radiol.* 58 353–361. 10.1177/0284185116649796 27273376

[B44] Nieuwenhuys R. (2012). The insular cortex: a review. *Prog. Brain Res.* 195 123–163. 22230626 10.1016/B978-0-444-53860-4.00007-6

[B45] Pannekoek J. N. Veer I. M. Van Tol M. J. Van Der Werff S. J. Demenescu L. R. Aleman A. (2013). Resting-state functional connectivity abnormalities in limbic and salience networks in social anxiety disorder without comorbidity. *Eur. Neuropsychopharmacol.* 23 186–195. 10.1016/j.euroneuro.2012.04.018 22749355

[B46] Paulesu E. Sambugaro E. Torti T. Danelli L. Ferri F. Scialfa G. (2010). Neural correlates of worry in generalized anxiety disorder and in normal controls: a functional MRI study. *Psychol. Med.* 40 117–124. 10.1017/S0033291709005649 19419593

[B47] Paulus M. P. Stein M. B. (2010). Interoception in anxiety and depression. *Brain Struct. Funct.* 214 451–463. 10.1007/s00429-010-0258-9 20490545 PMC2886901

[B48] Peterson A. Thome J. Frewen P. Lanius R. A. (2014). Resting-state neuroimaging studies: a new way of identifying differences and similarities among the anxiety disorders? *Can. J. Psychiatry* 59 294–300. 10.1177/070674371405900602 25007403 PMC4079145

[B49] Qiao J. Wang Z. Zhao G. Huo Y. Herder C. L. Sikora C. O. (2017). Functional neural circuits that underlie developmental stuttering. *PLOS ONE* 12:e0179255. 10.1371/journal.pone.0179255 28759567 PMC5536300

[B50] Qiao J. Weng S. Wang P. Long J. Wang Z. (2015). Normalization of intrinsic neural circuits governing Tourette’s syndrome using cranial electrotherapy stimulation. *IEEE Trans. Biomed. Eng.* 62 1272–1280. 10.1109/TBME.2014.2385151 25546850

[B51] Qin S. Young C. B. Duan X. Chen T. Supekar K. Menon V. (2014). Amygdala subregional structure and intrinsic functional connectivity predicts individual differences in anxiety during early childhood. *Biol. Psychiatry* 75 892–900. 10.1016/j.biopsych.2013.10.006 24268662 PMC3984386

[B52] Roy A. K. Fudge J. L. Kelly C. Perry J. S. Daniele T. Carlisi C. (2013). Intrinsic functional connectivity of amygdala-based networks in adolescent generalized anxiety disorder. *J. Am. Acad. Child Adolesc. Psychiatry* 52 290.e2–299.e2. 10.1016/j.jaac.2012.12.010 23452685 PMC3760686

[B53] Schienle A. Ebner F. Schafer A. (2011). Localized gray matter volume abnormalities in generalized anxiety disorder. *Eur. Arch. Psychiatry Clin. Neurosci.* 261 303–307. 10.1007/s00406-010-0147-5 20820793

[B54] Shin L. M. Liberzon I. (2010). The neurocircuitry of fear, stress, and anxiety disorders. *Neuropsychopharmacology* 35 169–191. 10.1038/npp.2009.83 19625997 PMC3055419

[B55] Slotnick S. D. (2017). Resting-state fMRI data reflects default network activity rather than null data: a defense of commonly employed methods to correct for multiple comparisons. *Cogn. Neurosci.* 8 141–143. 10.1080/17588928.2016.1273892 28002981

[B56] Slotnick S. D. Moo L. R. Segal J. B. Hart J. Jr. (2003). Distinct prefrontal cortex activity associated with item memory and source memory for visual shapes. *Brain Res. Cogn. Brain Res.* 17 75–82. 10.1016/S0926-6410(03)00082-X 12763194

[B57] Sorg C. Riedl V. Muhlau M. Calhoun V. D. Eichele T. Laer L. (2007). Selective changes of resting-state networks in individuals at risk for Alzheimer’s disease. *Proc. Natl. Acad. Sci. U.S.A.* 104 18760–18765. 10.1073/pnas.0708803104 18003904 PMC2141850

[B58] Strawn J. R. Wehry A. M. Chu W. J. Adler C. M. Eliassen J. C. Cerullo M. A. (2013). Neuroanatomic abnormalities in adolescents with generalized anxiety disorder: a voxel-based morphometry study. *Depress. Anxiety* 30 842–848. 10.1002/da.22089 23495075

[B59] Sylvester C. M. Corbetta M. Raichle M. E. Rodebaugh T. L. Schlaggar B. L. Sheline Y. I. (2012). Functional network dysfunction in anxiety and anxiety disorders. *Trends Neurosci.* 35 527–535. 10.1016/j.tins.2012.04.012 22658924 PMC3432139

[B60] Tye K. M. Prakash R. Kim S. Y. Fenno L. E. Grosenick L. Zarabi H. (2011). Amygdala circuitry mediating reversible and bidirectional control of anxiety. *Nature* 471 358–362. 10.1038/nature09820 21389985 PMC3154022

[B61] Wang W. Qian S. Liu K. Li B. Li M. Xin K. (2016). Reduced white matter integrity and its correlation with clinical symptom in first-episode, treatment-naive generalized anxiety disorder. *Behav. Brain Res.* 314 159–164. 10.1016/j.bbr.2016.08.017 27515289

[B62] Wang Z. Maia T. V. Marsh R. Colibazzi T. Gerber A. Peterson B. S. (2011). The neural circuits that generate tics in Tourette’s syndrome. *Am. J. Psychiatry* 168 1326–1337. 10.1176/appi.ajp.2011.09111692 21955933 PMC4246702

[B63] White S. F. Geraci M. Lewis E. Leshin J. Teng C. Averbeck B. (2017). Prediction error representation in individuals with generalized anxiety disorder during passive avoidance. *Am. J. Psychiatry* 174 110–117. 10.1176/appi.ajp.2016.15111410 27631963 PMC5572647

[B64] Zhang L. Zhang Y. Li L. Li Z. Li W. Ma N. (2011). Different white matter abnormalities between the first-episode, treatment-naive patients with posttraumatic stress disorder and generalized anxiety disorder without comorbid conditions. *J. Affect. Disord.* 133 294–299. 10.1016/j.jad.2011.03.040 21497403

[B65] Zhang Y. Li L. Yu R. Liu J. Tang J. Tan L. (2013). White matter integrity alterations in first episode, treatment-naive generalized anxiety disorder. *J. Affect. Disord.* 148 196–201. 10.1016/j.jad.2012.11.060 23305653

